# UBE2N plays a pivotal role in maintaining melanoma malignancy

**DOI:** 10.18632/oncotarget.26482

**Published:** 2018-12-21

**Authors:** Anushka Dikshit, Jennifer Y. Zhang

**Affiliations:** Department of Dermatology, Duke School of Medicine, Durham, NC, USA

**Keywords:** melanoma, K63-ubiquitination, UBE2N, MEK, FRA1

**Figure 1:**
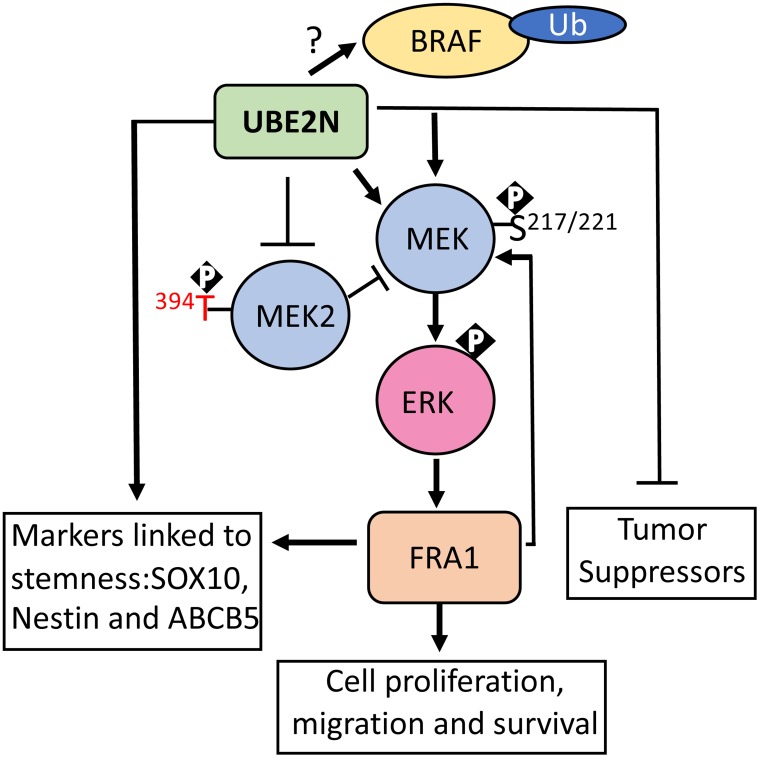
A schematic diagram of UBE2N functions in melanoma

Poly-ubiquitination is a post-translational modification that can impart diverse functions to its targets depending on the type of linkage [1]. K48-ubiquitination is primarily associated with proteasomal degradation, whereas K63-ubiquitination (K63-Ub) mainly regulates signal transduction and gene expression [1]. UBE2N is an E2 conjugating enzyme which acts through dimerization with a non-catalytic variant, either UBE2V1 or UBE2V2, to specifically catalyze the K63-Ub linkage [2]. UBE2N/UBE2V1 regulates the NF-κB and p38 signaling pathways and UBE2N/UBE2V2 stabilizes PCNA to support error-free DNA replication [3]. Recently, UBE2N has been characterized as a promising therapeutic target for breast cancer, neuroblastoma, and B-cell lymphoma [4–6]. In neuroblastoma and B-cell lymphoma, UBE2N prevents activation and in turn the nuclear translocation of p53 in addition to its well documented effect on stimulating NF-κB activation [4, 5]. In breast cancer, UBE2N acts by activating p38α through MAPK3 and TAK1 to induce metastasis [6]. Our studies reveal that UBE2N-Ub is up-regulated in melanoma cells, and plays a significant role in melanoma growth and tumor progression [7]. Inhibition of UBE2N either genetically via shRNA and CRISPR-mediated approaches or pharmacologically using NSC697923, a small molecule compound that disrupts UBE2N interaction with UBE2V1 and UBE2V2 [2], resulted in a markedly altered signaling landscape characterized by an attenuated MEK/ERK signaling cascade, decreased expression of markers linked to stemness such as FRA1, SOX10, ABCB5, and Nestin, and increased expression of differentiation markers such as MC1R, as well as cell senescent markers such as p16 and p53 [7]. Exogenous expression of a constitutively active FRA1 mutant prevented MEK inactivation and SOX10 downregulation. Our findings highlight a key role of UBE2N in promoting cell proliferation and prevention of cancer cell senescence, and characterized FRA1 as an important regulator of SOX10 and the MEK/ERK feed-forward signaling loop (Figure 1).

The BRAF/MEK/ERK signaling cascade is by far the most common oncogenic signaling pathway in melanoma. Specifically, BRAF is mutated in about 70% of cutaneous melanomas, leading to constitutive activation of MEK/ERK signaling. MEK inhibitors have produced impressive results, but their benefits are often short-lived, underscoring the need for new targeting strategies. We observed that inhibition of UBE2N significantly reduced expression of the activating phosphorylation of MEK1/2(S217/221) accompanied by an upregulation of the inhibitory phosphorylation of MEK2(T394) [7]. Interestingly, the effect of UBE2N loss on MEK was only observed in the BRAFV600E but not NRAS mutant melanoma cells, suggesting that UBE2N is especially important for BRAF-driven oncogenecity. It is previously reported that K63-Ub potentiates BRAF(V600E) oncokinase activity [8]. It remains to be addressed whether UBE2N supports MEK signaling via direct regulation of BRAF ubiquitination. By demonstrating UBE2N as a key regulator of MEK activation specifically in BRAF mutant cells, our findings indicate that UBE2N may be targeted for MEK inhibition in BRAF mutant cells.

In addition to the cancer cell-intrinsic effects, UBE2N plays a pivotal role in regulating immune and inflammatory pathways. Particularly, UBE2N is found to inhibit the conversion of regulatory T-lymphocytes to cytotoxic T-effector cells [9]. We have shown that systemic delivery of NSC697923 inhibits melanoma xenograft growth and malignancy in immunodeficient mice [7], indicating that UBE2N can be inhibited in vivo. Future studies are needed to determine whether UBE2N inhibition can modulate the tumor microenvironment and enhance anti-tumor immunity. Given the multifaceted functions of UBE2N in various cancers, it is possible that UBE2N specific inhibitors have broad therapeutic implications.
